# Imaging Findings of Jugular Foramen Meningocele in a Neurofibromatosis Type 1 Patient

**DOI:** 10.1155/2017/7047696

**Published:** 2017-12-24

**Authors:** Mehmet Serindere, Mustafa Tasar, Salih Hamcan, Ugur Bozlar

**Affiliations:** Gulhane Training and Research Hospital, Department of Radiology, University of Health Sciences, Ankara, Turkey

## Abstract

Neurofibromatosis type I (NF1) is a neurocutaneous disorder that involves autosomal dominant transmission. Skull defects, including sphenoid dysplasia and calvarial defects, are a rare finding in patients with NF1. Spinal meningocele and sphenoid wing dysplasia have been identified in NF1 but the occurrence of meningoceles at the skull base is extremely rare. A rare instance of jugular foramen meningocele being identified in an NF1 patient on imaging is described in this paper. To the best of our knowledge, only two such cases have been reported in the English literature.

## 1. Introduction

Neurofibromatosis type I (NF1) is a neurocutaneous disorder that involves autosomal dominant transmission. This disease is caused by a mutated NF1 gene on chromosome 17 and is characterized by inactive neurofibromin. The diagnostic criteria for NF1, with emphasis on the involvement of the skin, bones, and nervous system, was formulated at the National Institutes of Health consensus development conference [1]. Skull defects, including sphenoid dysplasia and calvarial defects, are a rare finding in patients with NF1. A rare instance of jugular foramen meningocele being identified in an NF1 patient on imaging is described in this paper.

## 2. Case Report

**Figure 1:**
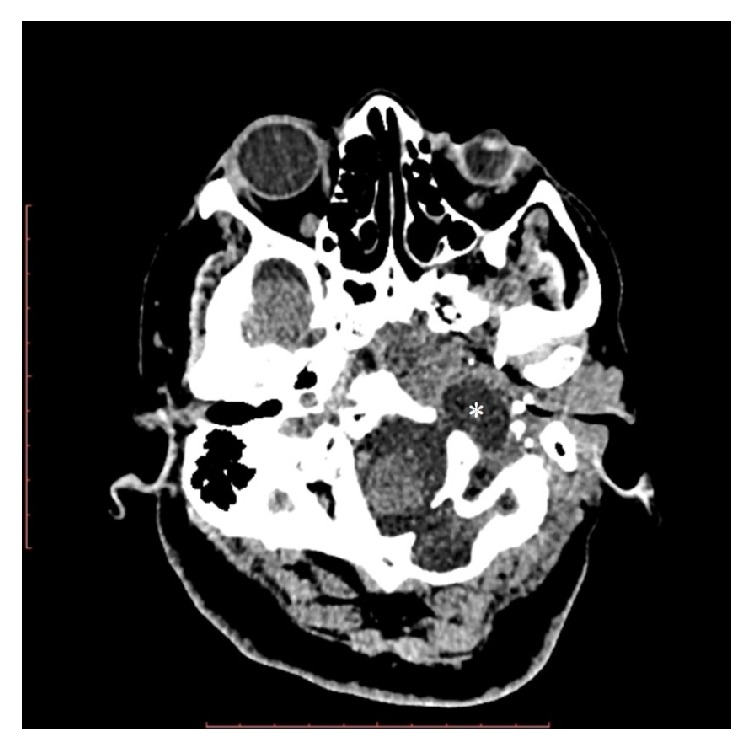
Axial CT image with bone window settings shows enlargement of left jugular foramen (*∗*).

**Figure 2:**
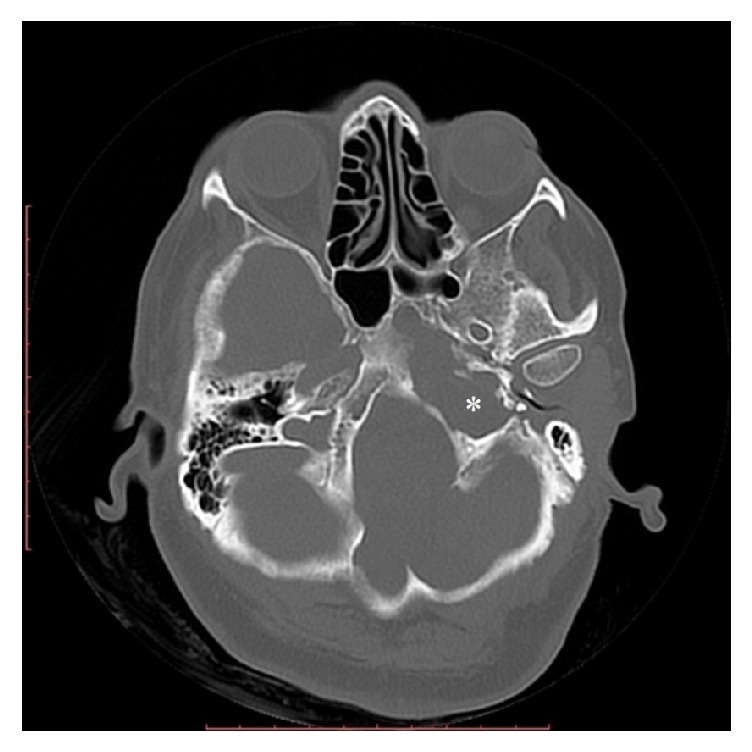
Axial CT image with the soft tissue window settings shows a low-density pouch herniated to the jugular foramen (*∗*).

A 59-year-old female patient with known NF1 presented at our institution with headache, without nausea or vomiting, of over a six-month duration. Multiple* café-au-lait* spots and axillary freckles were observed in the anterior and posterior aspect of the body during the physical examination. Focal neurological deficits and cranial nerve palsy were not found. The patient was referred for a computed tomography (CT) scan of the brain (Aquilion 64®; Toshiba Medical Systems, Otawara, Japan). Left jugular foramen enlargement was noticed, featuring a homogeneous, well defined, low-density lesion on CT imaging. The lesion had caused expansion in the adjacent bones (Figure 1). Its density was similar to that of cerebrospinal fluid (CSF) (Figure 2).

**Figure 3:**
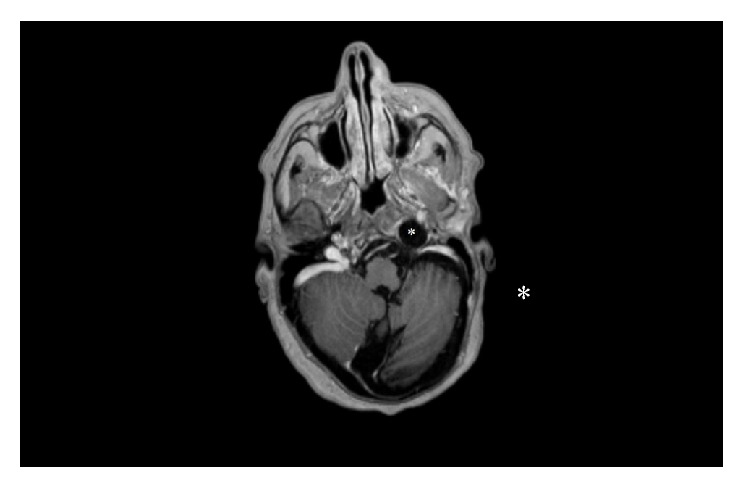
Axial T2 weighted image shows CSF and meninges herniated to the left jugular foramen (*∗*).

**Figure 4:**
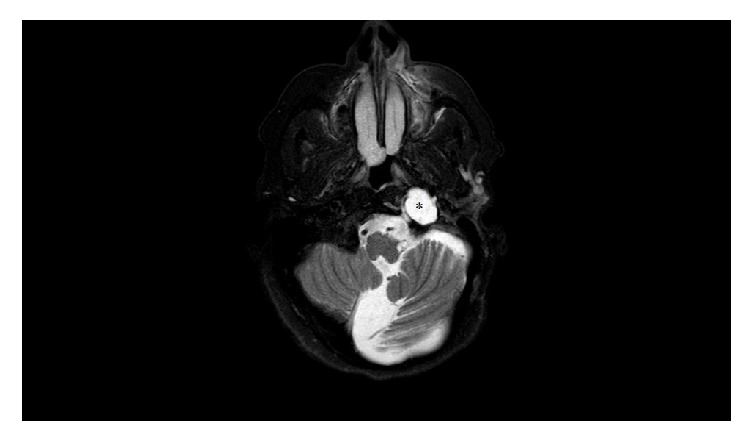
Axial postcontrast T1 weighted image shows CSF and meninges herniated to the left jugular foramen without enhancement (*∗*).

The patient underwent magnetic resonance imaging (MRI) (Achieva X-series®; Philips Healthcare, Best, the Netherlands) (using a 3.0 Tesla scanner) of the brain to evaluate the lesion. An enlarged left jugular foramen with a pouch was observed, extending from the left cerebellopontine angle cistern (Figure 3). This structure was isointense to the CSF signals in all sequences. Enhancement and diffusion restriction were not found following the administration of a gadolinium-based contrast agent (Figure 4), nor obvious herniations of brain parenchyma. Thus, the imaging findings were suggestive of meningocele in the left jugular foramen, identified incidentally. The laterally displaced jugular vein was compressed and the left internal carotid artery was displaced anteriorly by the mass effect of the meningocele. Annual follow-up appointments were recommended to determine progression and monitor potential enlargement of the defect over time.

## 3. Discussion

NF1 is an autosomal dominant neurocutaneous disorder (phakomatosis), with an incidence of roughly 1 per 3,000–4,000 live births [2]. It is characterized by cutaneous findings, most notably* café-au-lait* spots and axillary freckling, osseous dysplasia, and benign and malignant nervous system tumors, and neurofibromas in particular. Neurofibromas are benign tumors of the peripheral nerve sheath that are typically associated with NF1. Neurofibromas present as focal cutaneous/subcutaneous or nodular/diffuse plexiform lesions [1, 2].

Additional congenital malformation and syndromes (i.e., chromosomal anomalies, Dandy-Walker malformation, heterotopies, midline defects, and microcephaly) are also associated with cephaloceles. Several clinical manifestations, including meningitis caused by CSF rhinorrhea, cranial midline defects (i.e., cleft lip or cleft palate), and endocrine abnormalities, can also occur, depending on the size and location of the cephalocele [3]. Therefore, some patients without brain herniation, and in whom other syndromes are absent, can be asymptomatic until adulthood, as was the case with our patient.

Cephaloceles are divided into two groups: primary (congenital) and secondary. Primary cephaloceles may be occipital, parietal, sincipital, or basal. Sincipital cephaloceles are classified as nasofrontal, nasoethmoidal, nasoorbital, or combined and basal cephaloceles as transethmoidal, sphenoethmoidal, sphenoorbital, sphenomaxillary, and transsphenoidal. Secondary cephaloceles are sometimes caused by trauma or surgery [4].

Encephaloceles occur in approximately 1 in 3,000–5,000 live births [5]. Basal meningoencephaloceles are rare anomalies and have been reported to comprise 1–10% of all encephaloceles [6]. Spinal meningocele and sphenoid wing dysplasia have been found in NF1 but the occurrence of meningoceles at the skull base is extremely rare.

Jugular foramen involvement does not fit into the cephalocele classification. Indeed, only two case reports were identified in the literature at the time that this report was prepared [7, 8]. Siddiqui et al. [7] and Jeshil and Deepak [8] reported that jugular foramen meningocele was detected in adult patients with NF1 incidentally. The identification of jugular foramen meningocele in a neurofibromatosis type 1 patient using MRI and CT was described in this report.

## 4. Conclusion

This was an uncommon case of the identification of jugular foramen meningocele in a neurofibromatosis type 1 patient. As this pathology is so rare, it may not be recognized during the initial investigation of NF1 patients. Thus, physicians should be aware of this uncommon finding and of the importance of treating and managing such patients accordingly. Imaging findings and MRI in particular are obligatory for head and neck region neurofibromas and central nervous system manifestations in NF1 patients and also during follow-ups.
